# CA19.9 Response and Tumor Size Predict Recurrence Following Post-neoadjuvant Pancreatectomy in Initially Resectable and Borderline Resectable Pancreatic Ductal Adenocarcinoma

**DOI:** 10.1245/s10434-022-12622-w

**Published:** 2022-10-13

**Authors:** Laura Maggino, Giuseppe Malleo, Stefano Crippa, Giulio Belfiori, Sara Nobile, Giulia Gasparini, Gabriella Lionetto, Claudio Luchini, Paola Mattiolo, Marco Schiavo-Lena, Claudio Doglioni, Aldo Scarpa, Claudio Bassi, Massimo Falconi, Roberto Salvia

**Affiliations:** 1 Unit of General and Pancreatic Surgery, Department of Surgery and Oncology, University of Verona Hospital Trust, G.B. Rossi Hospital, Verona, Italy; 2 Unit of Pancreatic Surgery, Pancreas Translational and Clinical Research Center, San Raffaele Scientific Institute, Vita-Salute University, Milan, Italy; 3 Department of Diagnostics and Public Health, Section of Pathology, University of Verona Hospital Trust, Verona, Italy; 4 Division of Pathology, Pancreas Translational and Clinical Research Center, Vita-Salute University, San Raffaele Scientific Institute, Milan, Italy

## Abstract

**Background:**

Data on recurrence after post-neoadjuvant pancreatectomy are scant. This study investigated the incidence and pattern of recurrence in patients with initially resectable and borderline resectable pancreatic ductal adenocarcinoma who received post-neoadjuvant pancreatectomy. Furthermore, preoperative predictors of recurrence-free survival (RFS) and their interactions were determined.

**Patients and Methods:**

Patients undergoing post-neoadjuvant pancreatectomy at two academic facilities between 2013 and 2017 were analyzed using standard statistics. The possible interplay between preoperative parameters was scrutinized including interaction terms in multivariable Cox models.

**Results:**

Among 315 included patients, 152 (48.3%) were anatomically resectable. The median RFS was 15.7 months, with 1- and 3-year recurrence rates of 41.9% and 74.2%, respectively. Distant recurrence occurred in 83.3% of patients, with lung-only patterns exhibiting the most favorable prognostic outlook. Normal posttreatment CA19.9, ΔCA19.9 (both in patients with normal and elevated baseline levels), and posttreatment tumor size were associated with RFS. Critical thresholds for ΔCA19.9 and tumor size were set at 50% and 20 mm, respectively. Interaction between ΔCA19.9 and posttreatment CA19.9 suggested a significant risk reduction in patients with elevated values when ΔCA19.9 exceeded 50%. Moreover, posttreatment tumor size interacted with posttreatment CA19.9 and ΔCA19.9, suggesting an increased risk in the instance of elevated posttreatment CA19.9 values and a protective effect associated with CA19.9 response in patients with tumor size >20 mm.

**Conclusion:**

Recurrence following post-neoadjuvant pancreatectomy is common. Preoperative tumor size <20 mm, normal posttreatment CA19.9 and ΔCA19.9 > 50% were associated with longer RFS. These variables should not be taken in isolation, as their interaction significantly modulates the recurrence risk.

**Supplementary Information:**

The online version contains supplementary material available at 10.1245/s10434-022-12622-w.

The overall rate of recurrence following pancreatectomy for pancreatic ductal adenocarcinoma (PDAC) exceeds 80%,^1–3^ marking a clinically and emotionally critical time point in the disease trajectory.^4^ Observational data from the upfront-surgery setting has shown that nearly 60% of recurrences occur within a year postpancreatectomy, most commonly at distant sites, even after a margin-free resection.^1–3,5–7^ This led to an argument against the well-established surgery-first paradigm, providing the substrate for the recent implementation of a neoadjuvant treatment (NAT) approach. NAT has been proposed to extend the recurrence-free interval both directly, by ensuring better systemic disease control, and through a selection effect, enucleating patients with insufficient physiological resilience or aggressive tumor biology, who would have previously experienced poor results after surgery. Nonetheless, evidence on the incidence and characteristics of recurrence in patients who receive post-neoadjuvant pancreatectomy is scant.^8–10^ Moreover, posttreatment predictors of recurrence are ill defined, impairing a data-driven approach to surgical decision making.

With these issues in mind, the aim of this study was twofold: First, to investigate the incidence and pattern of recurrence in a large contemporary cohort of initially resectable and borderline resectable (BR) PDAC patients undergoing post-neoadjuvant pancreatectomy. Second, to determine posttreatment variables associated with recurrence-free survival (RFS), with particular regard to the possible interplay between various radiographic and biochemical parameters.

## Methods

### Study Design

After Institutional Review Board approval (PAD-R, n.1101CESC), patients undergoing post-neoadjuvant pancreatectomy for PDAC at the Unit of General and Pancreatic Surgery, University of Verona Hospital Trust, and at the Pancreatic Surgery Unit, San Raffaele University Hospital, Milan, from 2013 to 2017 were retrieved from prospectively maintained electronic databases. Resectability was classified according to the National Comprehensive Cancer Network (NCCN) guidelines^11^ and only patients who were resectable or BR at the time of diagnosis were included, in compliance with a rigorous definition of NAT.^11,12^ Additional exclusion criteria were distant metastases, macroscopically incomplete (R2) resection, in-hospital mortality, and missing information on recurrence or early censoring (<6 months, Study flowchart in Supplementary Fig. 1). Standard demographic, clinical, and surgical details were captured. Radiologic staging was integrated with the concepts of “biologic” and “conditional” BR disease, as proposed in the MD Anderson Cancer Center (MDACC) classification^13^ (Supplementary Table 1). Radiographic features and CA19.9 levels were assessed both at baseline and posttreatment. Tumor size was measured as the biggest diameter on computed tomography (CT) imaging, and radiographic response was assessed using the Response Evaluation Criteria in Solid Tumors (RECIST) v1 criteria.^14^ CA19.9 levels were considered evaluable only when the total bilirubin level was <2 U/mL. For patients experiencing jaundice at diagnosis (around 55% of the cohort), only CA19.9 values captured after endoscopic drainage (and subsequent bilirubin normalization—i.e., total bilirubin level <2 U/mL—were included in the analysis. When post-drainage CA19.9 values were not available, the data were considered as missing. The upper limit of normality used for CA19.9 was 37 U/ml. CA19.9 response was calculated as the percentage variation in response to NAT [ΔCA19.9** = **(baseline CA19.9 – posttreatment CA19.9)/baseline CA19.9]. Patients whose baseline levels were <5 U/mL were considered nonsecretors and analyzed as a separate group.^15^

### Patient Management

Throughout the study period, NAT was indicated for all BR patients and favored in anatomically resectable tumors exhibiting risk features (e.g., “biologic” and “conditional” BR tumors according to the MDACC classification).^13^ Chemotherapy regimens were assigned by the treating medical oncologist, and predominantly entailed FOLFIRINOX and gemcitabine + nab-paclitaxel. While the planned duration of NAT was 6 months in both institutions, the actual amount of chemotherapy depended on patient tolerance and radiological and biochemical response. Multidisciplinary evaluation of each case was performed following restaging. Minimum requirements for surgical eligibility were a stable disease per RECIST criteria and a performance status of 0–1 Eastern Cooperative Oncology Group (ECOG). Determinants of intraoperative resectability were absence of distant metastases, reconstructible superior mesenteric vein/portal vein, and no need for superior mesenteric artery resection. Pancreatectomies were performed in a standard fashion as previously described.^16^ Microscopic residual disease (R1) was determined based on the presence of tumor cells within 1 mm from any margin. The 8th Edition of the American Joint Committee of Cancer Staging Manual was applied.^17^ Active postoperative surveillance was carried out at 3–6-month intervals through physical examination, cross-sectional imaging, and measurement of CA19.9 serum levels. Disease recurrence was diagnosed radiographically in conjunction with clinical picture and/or CA19.9 levels; tissue diagnosis was occasionally performed. Follow-up was closed on July 2020.

### Outcome Measures

The RFS was computed from the date of surgery to the date of last follow-up or disease recurrence. For patients experiencing recurrence, the median post-recurrence survival (PRS) was evaluated, from the date of recurrence to the last follow-up. The location of first recurrence was classified as local (in the pancreatic remnant, resection bed, or along the peripancreatic vasculature), distant, or combined local and distant. Distant metastases were further classified based on the specific site (liver-only, lung-only, or multiple sites, including peritoneal carcinomatosis). The disease-specific survival (DSS) was calculated from the date of surgery to the date of last follow-up or disease-related death.

### Statistical Analysis

Data were analyzed using the R.4.0.0 software (Foundation for Statistical Computing, Vienna, Austria; https://www.r-project.org). Continuous variables were expressed as medians with interquartile range (IQR) and compared using Mann–Whitney *U* test. Categorical variables were presented as frequencies with percentages and compared using Chi-square or Fisher’s exact tests, as appropriate. All tests were two-tailed. Recurrence estimates were derived through life tables. Survival curves were constructed using the Kaplan–Meier method, and pairwise differences between groups were assessed using the log-rank test. While tumor size and CA19.9 parameters were initially handled as continuous variables, a minimum *p*-value approach was employed to identify clinically meaningful cut-off points. This entails selecting the threshold maximizing differences in RFS between groups. The association between clinically relevant preoperative variables and RFS was investigated through uni- and multivariable Cox regression models. The possible interplay between the various CA19.9 parameters and radiological features was investigated including interaction terms. A statistically significant interaction term indicates that the association of a given variable with RFS differs depending on the value of the covariate.^18^ The effect of the interaction was visualized plotting the conditional effects, which are the predictive values of one interaction term conditioned on certain (reference) levels of the other, using the *ggeffects* package. To avoid multicollinearity, different CA19.9 parameters were evaluated in distinct multivariable models. Other set of uni- and multivariable Cox regression models were also designed, including postoperative and pathologic data.

The amount of missing information for each variable accounted for less than 10% (Table 1). Preoperative data were considered to be missing at random and handled with multiple imputations with five permutations. Continuous variables were imputed by predictive mean matching, and binary variables by logistic regression. Pathologic, postoperative, and outcome variables (recurrence/survival) were not considered to be missing at random and were not imputed. The *p*-values are presented with odds ratios (OR) or hazard ratios (HR) and 95% confidence intervals (CI) as appropriate. Statistical significance was determined by a *p*-value <0.05.

**Table 1:** General characteristics and missing data of the study cohort (*n* = 315)

Variables	*n* (%) or median (IQR)	Missing, *n* (%)
Age at diagnosis, years	64.0 (57.0–70.0)	0 (0)
Sex		0 (0)
Male	140 (44.4)	
Female	175 (55.6)	
Body mass index	23.9 (21.7–26.6)	1 (0.3)
ASA score		0 (0)
1–2	222 (70.5)	
3–4	93 (29.5)	
Charlson age comorbidity index		0 (0)
<4	173 (54.9)	
≥4	142 (45.1)	
Diabetes		0 (0)
No	223 (70.8)	
Yes	92 (29.2)	
Circumstances of diagnosis		0 (0)
Incidental	64 (20.3)	
Symptoms	251 (79.7)	
Tumor location		0 (0)
Head	241 (76.5)	
Body-tail	74 (23.5)	
Resectability at diagnosis (NCCN)		0 (0)
Resectable	152 (48.3)	
Borderline resectable	163 (51.7)	
Baseline CA19.9, U/mL*	193.0 (63.7–669.0)	21 (6.7)
Tumor size at diagnosis, mm	30.0 (25.0–35.0)	22 (7.0)
MDACC class		0 (0)
Resectable	113 (35.9)	
A	129 (41.0)	
B	60 (19.0)	
C	13 (4.1)	
Type of neoadjuvant therapy		0 (0)
Chemotherapy	282 (89.5)	
Chemo–radiation	33 (10.5)	
Chemotherapy regimen		0 (0)
FOLFIRINOX	146 (46.3)	
Gemcitabine + nab-paclitaxel	137 (43.5)	
GEMOX	26 (8.3)	
Gemcitabine	6 (1.9)	
Attrition during NAT		0 (0)
No	277 (87.9)	
Yes	38 (12.1)	
Early NAT switch		0 (0)
No	298 (94.6)	
Yes	17 (5.4)	
Duration of chemotherapy (months)	4 (3–6)	0 (0)
Preoperative resectability (NCCN)		0 (0)
Resectable	206 (65.4)	
Borderline resectable	109 (34.6)	
RECIST response		14 (4.4)
Partial response	151 (50.2)	
Stable disease	150 (49.8)	
Preoperative CA19.9, U/mL**	30.0 (13.0–88.5)	35 (10.8)
Preoperative tumor size, mm	22.0 (18.0–30.0)	29 (9.2)
Type of surgery		0 (0)
Pancreaticoduodenectomy	220 (69.8)	
Distal pancreatectomy	52 (16.5)	
Total pancreatectomy	43 (13.7)	
Vascular resection		0 (0)
No	233 (74.0)	
Yes	82 (26.0)	
R-status		0 (0)
R0	197 (62.5)	
R1	118 (37.5)	
Lymph-vascular invasion		0 (0)
No	100 (31.7)	
Yes	215 (68.3)	
Perineural invasion		0 (0)
No	69 (21.9)	
Yes	246 (78.1)	
Peripancreatic fat invasion		0 (0)
No	94 (29.8)	
Yes	221 (70.2)	
T-Status		0 (0)
T1	127 (40.3)	
T2	146 (46.3)	
T3	18 (5.7)	
TX	24 (7.6)	
N-Status		0 (0)
N0	124 (39.4)	
N1	111 (35.2)	
N2	80 (25.4)	
Postoperative complications		0 (0)
No	135 (42.9)	
Yes	180 (57.1)	
Severe complications (Clavien–Dindo ≥ 3)		0 (0)
No	263 (83.5)	
Yes	52 (16.5)	
Adjuvant treatment		4 (1.3)
No	96 (30.9)	
Yes	215 (69.1)	
Adjuvant treatment type		
Chemotherapy only	111 (51.6)	0 (0)
Chemotherapy + radiation	85 (39.5)	
Radiation only	19 (8.8)	
Adjuvant chemotherapy regimen		
FOLFIRINOX	27 (13.8)	4 (2.0)
Gemcitabine + nab-paclitaxel	34 (17.3)	
Gemcitabine	89 (45.4)	
Capecitabine/5-fluorouracil	33 (16.8)	
Gemcitabine-capecitabine	4 (2.0)	
Other	5 (2.6)	

*ASA* American Society of Anesthesiologists; *NCCN*, National Comprehensive Cancer Network; *MDACC* MD Anderson Cancer Center

*Excludes CA19.9 nonsecretors. Median value is 144.5 U/mL (IQR 37.0–566.3) when non-secretors are included. **Excludes CA19.9 non-secretors. Median value is 25 U/mL (IQR 10–71.5) when nonsecretors are included. *NAT* neoadjuvant treatment

## Results

### Recurrence and Survival Outcomes

**Fig. 1:**
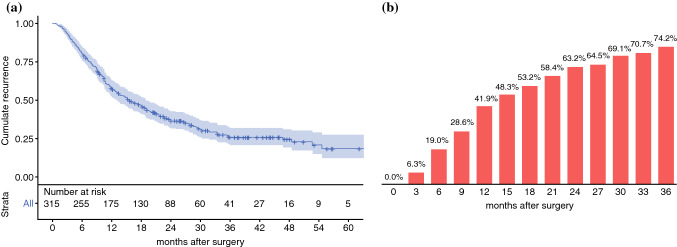
Kaplan–Meier survival curve of recurrence-free survival (**a**) and recurrence-estimates at various time-points after surgery (**b**)

**Fig. 2:**
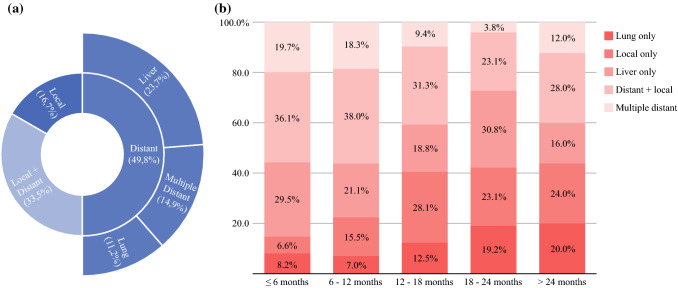
Overall pattern of recurrence of the study population (**a**) and stacked bar chart showing the proportion of recurrence patterns as a function of time elapsed after surgery (**b**)

The study population consisted of 315 patients, of whom 152 (48.3%) were anatomically resectable at diagnosis. Their characteristics are presented in Table 1. The median follow-up was 24.9 months (IQR 33.3–13.8 months) from surgery and 33.3 months (IQR 24.1–45.2 months) from diagnosis. At the time of the last contact, 166 patients (52.7%) were still alive, with a median follow-up of 30.8 months from surgery (IQR 20.9–43.2 months) and 39.8 months from diagnosis (IQR 29.8–50.4 months). The median RFS was 15.7 months (95% CI 12.7–18.7 months) (Fig. 1a). Disease recurrence manifested in 215/315 patients (68.3%). The estimated recurrence rate exceeded 40% at 1-year postoperatively and approached 75% at 3-years postoperatively (Fig. 1b). Isolated local recurrence occurred in 16.7% of patients (*n* = 36), distant metastases in 49.8% (*n* = 107) and combined recurrence in 33.5% (*n* = 72) of cases (Fig. 2a). The proportion of recurrence location as a function of time from pancreatectomy is shown in Fig. 2b. The median postoperative DSS was 41.3 months (95% CI 35.0–47.5 months). Survival outcomes varied depending on the specific recurrence pattern, with lung-only and multiple-distant sites exhibiting the most and least favorable prognostic outlook, respectively (Table 2 and Supplementary Fig. 2).

**Table 2:** Median disease-specific survival (DSS), recurrence-free survival (RFS), and post-recurrence survival (PRS) stratified by recurrence site (*n* = 215)

Recurrence site	*n* (%)	Median DSS	Median RFS	Median PRS
Overall		28.7 (25.2–32.2)	10.2 (9.2–11.2)	11.5 (8.8–14.2)
Local-only	36 (16.7)	28.9 (11.7–46.2)	13.5 (10.8–16.2)	11.5 (5.3–17.6)
Liver-only	51 (23.7)	25.6 (15.3–35.9)	9.4 (7.9–10.8)	10.8 (7.3–14.3)
Lung-only	24 (11.2)	37.4 (29.2–45.6)	12.1 (2.5–21.8)	19.8 (8.3–31.3)
Multiple distant	32 (14.9)	20.1 (15.4–24.7)	8.2 (6.3–10.0)	6.8 (3.0–10.5)
Local+ distant	72 (33.5)	27.7 (21.9–33.4)	8.9 (7.5–10.3)	13.9 (7.8–19.9)
*p*-value		Overall: 0.031	Overall: 0.069	Overall: 0.159
		Local-only versus multiple distant: 0.019 Lung-only versus liver-only: 0.050 Lung-only vesus multiple distant: 0.016 Lung-only versus distant + local: 0.045	Local-only versus multiple distant: 0.037 Local-only versus local + distant: 0.020 Lung-only versus local + distant: 0.024	Lung-only versus multiple distant: 0.035

### Radiologic Parameters and RFS

The median RFS was not significantly different based on resectability status, either at baseline (16.3 vs 14.3 months for resectable and borderline resectable patients, *p* = 0.318) or posttreatment (15.7 vs 14.3 months, *p* = 0.233). RECIST response was indeed associated with RFS (20.0 months vs 12.7 months for partial response vs stable disease, *p* = 0.002). Tumor size, analyzed as a continuous variable, was not associated with RFS at baseline (HR 1.004, 95% CI 0.995–1.013, *p* = 0.364), yet turned to be significant on posttreatment evaluation (HR 1.037, 95% CI 1.023–1.052, *p* < 0.001). Differences in RFS were maximized by a threshold of 19 mm (*p* = 7.34 × 10^−7^). Rounding this to 20 mm, 170/315 patients had a tumor size above the threshold (54.0%), with RFS being 25.0 versus 10.8 months for ≤20 mm versus >20 mm (Supplementary Fig. 3). Stratified analyses by tumor size are presented in Table 3. Notably, RECIST response did not remain statistically significant, while a posttreatment tumor size >20 mm was significantly associated with RFS in both the partial response and stable disease groups.

**Table 3:** Stratified analysis of the association between radiological and CA19.9 parameters, and recurrence-free survival (RFS)

		Total *n* (%)	Median RFS Months (95% CI)	*p*-value
*Radiological parameters*				
	RECIST response			
Posttreatment tumor size				
≤20 mm	Partial response	108 (74.5)	27.0 (21.4–32.7)	0.424
	Stable disease	37 (25.5)	19.0 (13.9–24.2)	
>20 mm	Partial response	52 (30.6)	11.7 (8.4–14.9)	0.388
	Stable disease	118 (69.4)	10.8 (8.7–12.8)	
	Posttreatment tumor size			
RECIST response				
Partial response	≤20 mm	108 (67.5)	27.0 (21.4–32.7)	**0.004**
	>20 mm	52 (32.5)	11.7 (8.4–14.9)	
Stable disease	≤20 mm	37 (23.9)	19.0 (13.9–24.2)	**0.012**
	>20 mm	118 (76.1)	10.8 (8.7–12.8)	
CA19.9 parameters*				
	Baseline CA19.9			
Posttreatment CA19.9				
Normal	Normal	40 (24.7)	19.0 (10.3–27.8)	0.523
	Elevated	122 (75.3)	17.6 (12.5–22.7)	
Elevated	Normal	9 (7.3)	5.2 (2.8–7.5)	**0.014**
	Elevated	114 (92.7)	11.8 (9.2–14.0)	
Delta CA19.9 ≥50%				
No	Normal	38 (44.2)	13.5 (10.0–17.0)	0.288
	Elevated	48 (55.8)	9.3 (6.6–12.0)	
Yes	Normal	11 (5.5)	NR	0.086
	Elevated	188 (94.5)	16.2 (11.6–20.7)	
	Posttreatment CA19.9			
Baseline CA19.9				
Normal	Normal	40 (81.6)	19.0 (10.3–27.8)	**0.002**
	Elevated	9 (18.4)	5.2 (2.8–7.5)	
Elevated	Normal	122 (51.7)	17.6 (12.5–22.7)	0.132
	Elevated	114 (48.3)	11.8 (9.2–14.0)	
Delta CA19.9 ≥50%				
No	Normal	32 (37.2)	14.8 (10.1–19.4)	0.078
	Elevated	54 (62.8)	9.1 (6.3–11.9)	
Yes	Normal	130 (65.3)	18.9 (14.2–23.6)	0.507
	Elevated	69 (34.7)	14.7 (10.7–18.7)	
	Delta CA19.9 ≥ 50%			
Baseline CA19.9				
Normal	No	38 (77.6)	13.5 (10.0–17.0)	**0.032**
	Yes	11 (22.4)	NR	
Elevated	No	48 (20.3)	9.3 (6.6–12.0)	**0.001**
	Yes	188 (79.7)	16.2 (11.6–20.7)	
Posttreatment CA19.9				
Normal	No	32 (19.8)	14.8 (10.1–19.4)	0.262
	Yes	130 (80.2)	18.9 (14.2–23.6)	
Elevated	No	54 (43.9)	9.1 (6.3–11.9)	**0.006**
	Yes	69 (56.1)	14.7 (10.7–18.7)	

Bold value indicates statistical significance (*p* < 0.05)

*n = 285 (30 CA19.9 non-expressors excluded)

### CA19.9 Serum Levels and RFS

Baseline CA19.9 levels were not significantly associated with RFS (16.3, 14.3, and 29.1 months for normal, elevated, and not expressed, respectively, *p* = 0.120), while there were significant differences in RFS based on posttreatment CA19.9 (17.7, 11.5, and 29.1 months for normal, elevated, and not expressed, respectively, *p* = 0.009). After excluding CA19.9 nonsecretors (*n* = 30), ΔCA19.9 was significantly associated with RFS (HR 0.992, 95% CI 0.985–0.999, *p* = 0.023), with a critical value maximizing RFS differences set at 53.8% (*p* = 7.26 × 10^−4^), which was approximated at 50.0%. Based on this definition, 199 patients (69.8%) experienced a CA19.9 response, while 86 (30.2%) were nonresponders, with RFS being 17.7 months in the former group and 11.5 months in the latter (Supplementary Fig. 4). On stratified analyses, an elevated posttreatment CA19.9 was associated with a shorter RFS only in the cohort of patients with normal baseline values, and was not a significant predictor of RFS when stratifying patients by CA19.9 response (Table 3). Conversely, ΔCA19.9 was significantly associated with RFS both in patients with normal and elevated baseline CA19.9 values. Moreover, ΔCA19.9 remained significantly associated with RFS in patients with elevated levels, but not in those with normal posttreatment CA19.9 values.

### Predictors of RFS—Preoperative Model

**Fig. 3:**
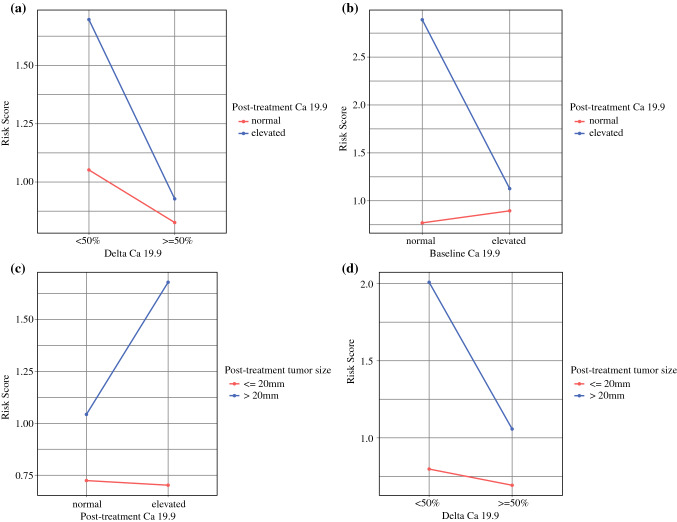
Graphical depiction of the interaction between posttreatment CA19.9 and delta CA19.9 (**a**), posttreatment CA19.9 and baseline CA19.9 (**b**), posttreatment tumor size and CA19.9 (**c**), and posttreatment tumor size and delta CA19.9 (**d**)

The uni- and multivariable analyses of preoperative variables associated with RFS in the study cohort are presented in Table 4. Tumor location (tail), duration of chemotherapy, elevated posttreatment CA19.9, and posttreatment tumor size were associated with shorter RFS. Indeed, ΔCA19.9 ≥ 50% was associated with longer RFS. When including interaction terms in the model, in the cohort of CA19.9 expressors, a significant interaction was confirmed between ΔCA19.9 and posttreatment CA19.9 (HR 0.551, 95% CI 0.364–0.835, *p* = 0.005), suggesting a significant risk reduction in patients with elevated posttreatment CA19.9 values, when ΔCA19.9 exceeded 50% (Fig. 3a). Moreover, the interaction between baseline and posttreatment CA19.9 was also significant (HR 0.371, 95% CI 0.217–0.453, *p* = 0.020), indicating a particularly elevated risk in the instance of CA19.9 elevation during NAT (Fig. 3b).

**Table 4:** Uni- and multivariable analysis of factors associated with recurrence-free survival in the study cohort (*n* = 315)

	Univariable analysis		Multivariable analysis	
	Hazard ratio (95% CI)	*p*-value	Hazard ratio (95% CI)	*p*-Value
Age at diagnosis, years	1.010 (0.996–1.025)	0.154		
Sex				
Male	1 (ref)	–		
Female	1.055 (0.806–1.382)	0.695		
Body mass index	1.003 (0.969–1.040)	0.850		
ASA score				
1–2	1 (ref)	–		
3–4	1.239 (0.926–1.658)	0.148		
Charlson age comorbidity index				
<4	1 (ref)	–		
≥4	1.095 (0.837–0.431)	0.508		
Diabetes				
No	1 (ref)	–		
Yes	1.022 (0.761–1.371)	0.886		
Circumstances of diagnosis				
Incidental	1 (ref)	–		
Symptoms	0.917 (0.658–1.278)	0.609		
Tumor location				
Head	1 (ref)	–	1 (ref)	–
Body-tail	**1.537 (1.134–2.082)**	**0.006**	**1.527 (1.119–2.083)**	**0.008**
Resectability at diagnosis (NCCN)				
Resectable	1 (ref)	–		
Borderline resectable	1.141 (0.872–1.493)	0.335		
Serum CA19.9 at diagnosis^+°^	0.996 (0.990–1.001)	0.137		
Serum CA19.9 at diagnosis^+^				
Normal (≤37 U/mL)	1 (ref)	–		
Elevated (>37 U/mL)	1.059 (0.729–1.539)	0.762		
Not expressed	0.603 (0.323–1.127)	0.113		
Tumor size at diagnosis, mm	1.004 (0.995–1.013)	0.364		
MDACC class				
Resectable	1 (ref)	–		
A	1.058 (0.766–1.443)	0.721		
B	1.125 (0.770–1.642)	0.543		
C	1.307 (0.674–2.534)	0.429		
Type of neoadjuvant therapy				
Chemotherapy	1 (ref)	–		
Chemoradiation	0.929 (0.597–1.444)	0.742		
Chemotherapy regimen				
FOLFIRINOX	1 (ref)	–		
Gemcitabine + nab– paclitaxel	1.142 (0.858–1.520)	0.363		
GEMOX	1.120 (0.685–1.831)	0.652		
Gemcitabine	1.997 (0.874–4.563)	0.101		
Attrition during neoadjuvant therapy				
No	1 (ref)	–		
Yes	1.065 (0.711–1.595)	0.759		
Duration of chemotherapy (months)	**1.094 (1.032–1.172)**	**0.011**	**1.126 (1.047–1.211)**	**0.001**
Early neoadjuvant therapy switch				
No	1 (ref)	–		
Yes	**1.602 (1.292–1.987)**	**<0.001**		
Preoperative resectability (NCCN)				
Resectable	1 (ref)	–		
Borderline resectable	1.176 (0.891–1.553)	0.253		
RECIST response				
Partial response	1 (ref)	**–**		
Stable disease	**1.512 (1.155–1.980)**	**0.003**		
Time from diagnosis to surgery	1.015 (0.976–1.054)	0.462		
Preoperative CA19.9 Serum levels^+°^	1.011 (0.994–1.028)	0.226		
Preoperative CA19.9 Serum levels^+^ levels^+^				
Normal (≤37 U/mL)	1 (ref)	–	1 (ref)	–
Elevated (>37 U/mL)	**1.385 (1.048–1.831)**	**0.022**	**1.391 (1.049–1.844)**	**0.022**
Not expressed	0.657 (0.376–1.145)	0.138	0.706 (0.404–1.233)	0.221
Delta CA19.9*^+^	**0.992 (0.985–0.999)**	**0.023**	**0.991 (0.984–0.998)**	**0.018**
Delta CA19.9 ≥ 50%^+^				
No	1 (ref)	–	1 (ref)	–
Yes	**0.615 (0.458–0.825)**	**0.001**	**0.640 (0.475–0.863)**	**0.003**
Not expressed	**0.405 (0.228–0.721)**	**0.002**	**0.450 (0.252–0.801)**	**0.007**
Preoperative tumor size, mm^#^	**1.037 (1.023–1.052)**	**<0.001**	**1.033 (1.019–1.047)**	**<0.001**
Preoperative tumor size^#^				
≤20 mm	1 (ref)	–	1 (ref)	–
>20 mm	**1.929 (1.463–2.542)**	**<0.001**	**2.224 (1.603–3.085)**	**0.021**

Bold value indicates statistical significance (*p* < 0.05)

*ASA* American Society of Anesthesiologists, *NCCN* National Comprehensive Cancer Network, *MDACC* MD Anderson Cancer Center

*Non-expressors excluded (total *n* = 285). ^+^To avoid collinearity, these variables were analyzed in mutually exclusive multivariable models. ^#^To avoid collinearity, these variables were analyzed in mutually exclusive multivariable models. °Hazard Ratios refer to a 100 U/mL unitary increase

When combining radiologic and CA19.9 parameters, posttreatment tumor size was found to significantly interact with both posttreatment CA19.9 (HR 1.619, 95% CI 1.134–2.310, *p* = 0.008, Fig. 3c) and ΔCA19.9 (HR 0.566, 95% CI 0.392–0.817, *p* = 0.002, Fig. 3d). This suggests an increased risk in the instance of an elevated posttreatment CA19.9, and a protective effect associated with CA19.9 response in the cohort of patients with greater tumor size. Conversely, baseline CA19.9 did not significantly interact with tumor size.

### Predictors of RFS–Postoperative Model

The analysis of pathologic and clinical factors associated with RFS is presented in Supplementary Table 2. After multivariable adjustment, R-status (HR 1.350, 95% CI 1.001–1.821, *p* = 0.049) together with AJCC T-status (HR 1.550, 95% CI 1.093–2.197, *p* = 0.014 for ypT2 vs ypT1; HR 2.200, 95% CI 1.202–4.028, *p* = 0.011 for ypT3 vs ypT1) and N-status (HR 1.127, 95% CI 0.791–1.605, *p* = 0.509 for ypN1 vs ypN0; HR 2.244, 95% CI 1.517–3.317, *p* < 0.001 for ypN2 vs ypN0) remained independent predictors of RFS.

## Discussion

This bi-institutional effort offers novel insight into the dynamics and predictors of recurrence following post-neoadjuvant pancreatectomy in initially resectable and BR-PDAC. Recurrence was a common event, manifesting relatively early in the postresection history, with a median RFS of 15.7 months and estimated recurrence rates approximating 40% at 1 year and 75% at 3 years. Previous studies including potentially resectable patients showed mixed outcomes. In an observational analysis from the Medical College of Wisconsin, 61% of patients developed recurrence and 55% of those who recurred were found with recurrent PDAC within 1 year from the operation.^8^ In the PACT-15 randomized trial, the per-protocol median event-free survival in the NAT arm (cisplatin, epirubicin, gemcitabine, and capecitabine) was 16.9 months,^19^ while the RFS in the NAT arm (gemcitabine-based chemoradiotherapy) of the multicenter, randomized PREOPANC-1 trial was only 8.1 months.^20^ Last, the recent SWOG 1505 trial showed similar recurrence outcomes in FOLFIRINOX (median RFS of 10.9 months) and the gemcitabine + nab-paclitaxel arm (median RFS of 14.2 months).^21^ Even in the present analysis, the value of chemotherapy and complementary radiation therapy as surrogate endpoints for recurrence could not be proven.

Notably, most patients experienced distant failure, with the incidence of isolated local recurrence being only around 16%, as already reported in the upfront surgery setting.^6,22^ This indicates that disease control offered by NAT is at best temporary, as viable micrometastatic clones can persist after systemic treatment and resection. While this is to some extent sobering, a positive impact of NAT relative to upfront pancreatectomy would likely become evident, accounting for the immortal time equal to the duration of preoperative treatment.

Moreover, this analysis confirms that distinct recurrence patterns are associated with unique prognostic profiles.^9^ In particular, recurrence at multiple distant sites had a daunting prognosis, with a median PRS of only 6.8 months. Conversely, in the rare instance of isolated lung relapse, the median DSS was as high as 37.4 months, with a median PRS of 19.8 months. These findings confirm the relatively indolent nature of pulmonary metastases even in the post-NAT scenario.

A further aim of this study was to determine posttreatment variables associated with RFS. Notwithstanding the increased use of NAT, the optimal metrics of treatment response remain nebulous, with the patient selection process for surgical exploration varying substantially between surgeons and institutions.^23^ As a general principle, surgical exploration is virtually always recommended when the patient is fit, and the disease is stable, at least biochemically and radiologically.^11,19^ While radiological downstaging seems to be a poor efficacy surrogate,^24–26^ certain parameters, such as tumor size or the percentage variation in tumor volume have been proposed to be of some value.^27,28^ In the present series, the median RFS was not significantly different stratifying by resectability status, neither at baseline nor posttreatment. Similarly, RECIST response was not associated with RFS. Conversely, tumor size was associated with RFS irrespective of RECIST response, with a posttreatment cut-off of 2 cm maximizing survival differences between groups. Although gross residual tumor after NAT might be a surrogate marker of poor treatment response, this approach remains somewhat unrefined. Looking forward, radiomics has shown early promise in exploiting the latent information present in radiological images and linking quantitative imaging biomarkers with response to systemic therapy.^29^

Evidence on serum CA19.9 has been more consistent, even though proposed variations on this theme have been the most diverse, with baseline posttreatment levels and/or their trend being variously associated with patient outcomes.^15,30,31^ Altogether, there is no agreement on how to exactly assess biochemical response. Most available studies define the optimal CA19.9 response as the presence of normal values posttreatment,^8,27,32^ while in other cases, a cut-off of 100 U/mL was arbitrarily introduced.^9,28^ Because these approaches neglect a patient’s history before the preoperative period, dynamic measures might be better suited to provide a comprehensive picture of the degree of treatment response. In this respect, some reports have shown that a decline in CA19.9 levels greater than 50% was an independent predictor of postresection survival.^10,33,34^ By contrast, in a series of 131 posttreatment pancreatectomies, Tsai et al. reported that only posttreatment normal CA19.9 values, but not the magnitude of its change (expressed as quartiles of ΔCA19.9), was an independent prognostic factor.^35^ We built on these reports by utilizing the same dynamic approach to ΔCA19.9 calculation, trying to determine the optimal cut-off and explore the interplay between the various interpretations of CA19.9 response. Both preoperative normal values and the percentage variation during treatment (with an optimal cut-off set at 50%) were independently associated with RFS. Moreover, a significant interaction was confirmed between ΔCA19.9 and posttreatment CA19.9, suggesting a substantial risk reduction in patients with elevated posttreatment CA19.9 values, when the ΔCA19.9 exceeded 50%. The analysis was finally compounded by the demonstration that posttreatment tumor size significantly interacts with both posttreatment CA19.9 and ΔCA19.9, in a complex relationship of reciprocal risk modulation. This emphasizes the need to evaluate treatment response parameters dynamically and in their mutual relationships.

Adjusted analysis showed that chemotherapy duration was associated with shorter DFS. Defining optimal NAT duration in patients with potentially resectable PDAC is an unmet need, but is beyond the scope of this retrospective analysis. Assessment of treatment activity over time has been previously performed using time to CA19.9 nadir as a measure of disease control.^36^ Because time to CA19.9 nadir was found to occur between 4 and 6 months in approximately 75% of patients, it has been suggested that this treatment timeframe is the most suitable in localized disease. Although in the present study 25% of patients received chemotherapy for more than 6 months, the observed trend towards a worse DFS for longer treatment programs is likely the result of a selection bias. Indeed, favorable anatomical/biochemical characteristics after first restaging prompted immediate surgery in the absence of a prespecified treatment duration plan for most patients. Remarkably, this contrasts with results from a series of BR/LA patients undergoing total neoadjuvant therapy and surgical resection at the Mayo Clinic, showing that extended duration of chemotherapy (defined as >6 cycles) was associated with improved survival.^26^

The present study provides a reference for recurrence estimates in patients undergoing pancreatectomy after a properly defined neoadjuvant strategy. We believe that RFS—rather than overall survival—is a more proximate expression of the degree of disease control achieved by surgical resection, limiting the possible confounding due to recurrence treatment. Moreover, these results emphasize the relationship between recurrence, tumor size and biochemical response, expressed not only as CA19.9 normalization, but also as the magnitude of its change. Most importantly, the concept of the dynamic interaction between response metrics was introduced, possibly providing a new paradigm for the analysis of survival data. Finally, knowledge of the patient risk profile relative to recurrence might guide the postresection surveillance and the interpretation of indeterminate radiographic findings or isolated CA19.9 elevation, as establishing early salvage chemotherapy has the potential to improve survival.^1^

This study has also limitations. First, the denominator is represented by all patients who made it for resection. As such, results cannot be extrapolated to the overall cohort of potentially resectable patients who experienced presurgical and intraoperative attrition.^37^ Second, criteria for chemotherapy regimen selection and triage of surgical candidates reflects the practice of two academic institutions specialized in pancreatic surgery, possibly limiting the generalizability of these results to low-volume centers. Third, information on the treatment strategy adopted for recurrence was not uniformly available, so that its direct impact could not be investigated. Finally, some subgroup analyses might be underpowered to reveal significant differences between groups.

In summary, this bi-institutional analysis of initially resectable and BR patients receiving post-neoadjuvant pancreatectomy showed a median RFS of 15.9 months, with 1- and 3-year recurrence estimates of 41.9% and 74.2%, respectively. In the framework of a real-world practice, a preoperative tumor size <2 cm, normal posttreatment CA19.9 values, and ΔCA19.9 > 50% were associated with longer RFS. Importantly, these variables should not be taken in isolation, as their interaction significantly modulates the recurrence risk.

## Supplementary Information

Below is the link to the electronic supplementary material.

Supplementary file1 (DOCX 546 kb)

Supplementary file2 (DOCX 20 kb)
