# Supplementary material for: CA19.9 Response and Tumor Size Predict Recurrence Following Post-neoadjuvant Pancreatectomy in Initially Resectable and Borderline Resectable Pancreatic Ductal Adenocarcinoma
Source: Ann Surg Oncol. 2022 Oct 13;30(1):207–19. doi: 10.1245/s10434-022-12622-w (PMC9726670; doi:10.1245/s10434-022-12622-w)
Supplement: Supplementary file 1 — Supplementary file1 (DOCX 546 kb) [file 10434_2022_12622_MOESM1_ESM.docx]

**Supplementary Figure 1.** Study flow-chart

Surgical exploration after primary chemotherapy for PDAC (2013-2017)

n=554

Locally advanced or metastatic at diagnosis n=122 (22.0%)

Non-curative-intent surgery n=7 (1.3%)

Non–resected patients n=56 (10.1%)

Study population

n=315

Patients eligible to recurrence analysis

n=350

Resection after neoadjuvant treatment for PDAC (2013-2017)

n=369

Metastatic patients n=10 (2.3%)

R2 Resections n=4 (0.9%)

Postoperative deaths n=5 (1.2%)

Missing information on recurrence n=35 (10.0%)

**Supplementary Figure 2.** Kaplan-Meier curves of disease-specific survival (a), recurrence-free survival (b) and post-recurrence survival (c) of patients experiencing recurrence stratified by recurrence pattern.

**
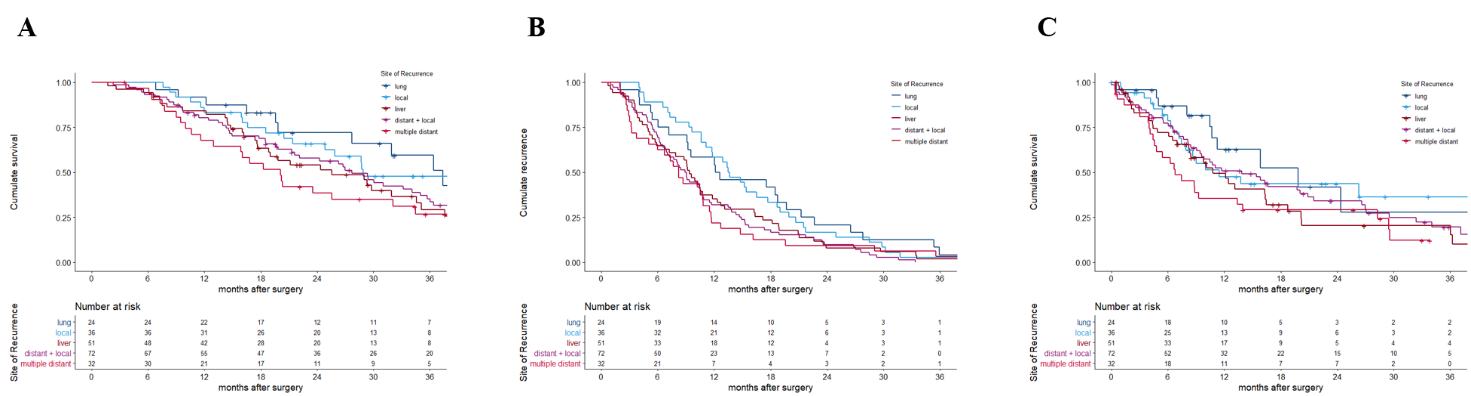
**

**Supplementary Figure 3.** Kaplan-Meier curves stratified by RECIST response (a) and post-treatment tumor size (b).


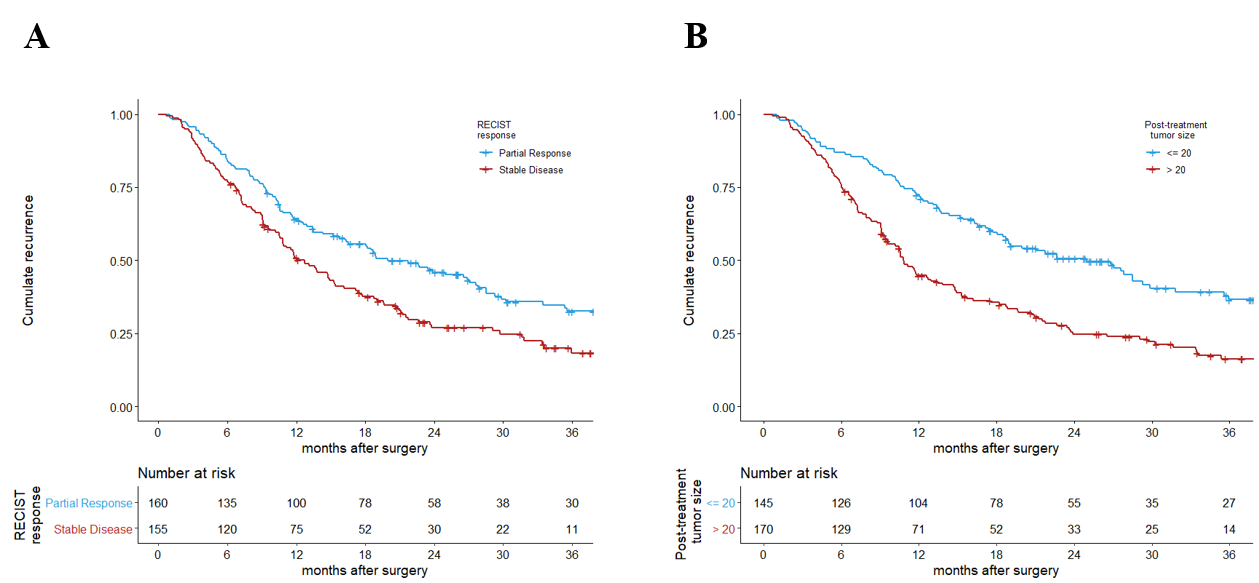


**Supplementary Figure 4.** Kaplan-Meier curves stratified by baseline Ca 19.9 (a), post-treatment Ca 19.9 (b) and delta Ca 19.9 (c)

**
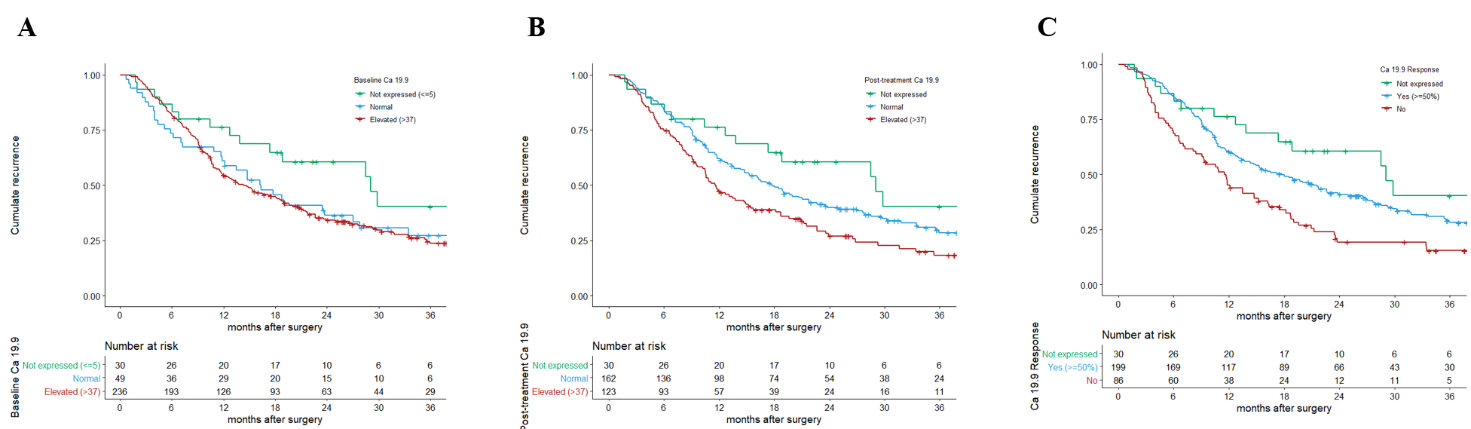
**
