# Supplementary material for: CA19.9 Response and Tumor Size Predict Recurrence Following Post-neoadjuvant Pancreatectomy in Initially Resectable and Borderline Resectable Pancreatic Ductal Adenocarcinoma
Source: Ann Surg Oncol. 2022 Oct 13;30(1):207–19. doi: 10.1245/s10434-022-12622-w (PMC9726670; doi:10.1245/s10434-022-12622-w)
Supplement: Supplementary file 2 — Supplementary file2 (DOCX 20 kb) [file 10434_2022_12622_MOESM2_ESM.docx]

**Supplementary Table 1.** MD Anderson Cancer Center (MDACC) classification of borderline resectable disease.

| **A** | **Patients with borderline resectable tumor anatomy as defined on cross-sectional imaging to include one or more of the following:**  - Tumor abutment (≤180°) of the superior mesenteric artery or celiac axis  - Tumor abutment or encasement (>180°) of a short segment of the hepatic artery  - Short-segment occlusion of the superior mesenteric vein and/or the portal vein amenable to resection and reconstruction |
| --- | --- |
| **B** | **Patients with borderline resectable disease owing to a concern for possible extrapancreatic metastatic disease. This includes:**  - Imaging findings suspicious for, but not diagnostic of, metastatic disease (e.g. indeterminate liver lesions or distant lymph nodes)  - Cytologically/histologically proven metastasis in regional lymph nodes  - Ca 19.9 > 1000 U/mL with a normal total bilirubin |
| **C** | **Patients with borderline resectable disease owing to age, comorbidities, or poor performance status^*^. These conditions include:**  - Age ≥ 80  - Comorbid conditions requiring workup and evaluation before surgery (chronic obstructive pulmonary disease, myocardial infarction in the last 6 months or angina in past month, stroke or transient ischemia history, steroid use in past month, ascites or varices, and/or preoperative sepsis or inflammatory condition)  - Marginal performance status (ECOG ≥2) expected eventually to improve |

MDACC type-C takes priority over type-B when patients have characteristics of both.

^*^Although present in the original MDACC classification, weight loss >10%in the last 6 months is not considered as a criterion for type-C BR disease in our institutional practice, due to its common occurrence

**Supplementary Table 2.** Clinical and pathological variables associated with recurrence-free survival.

| **Variables**  **n (%)** | **315 (100)** | **Univariable analysis** | | **Multivariable analysis^*^** | |
| --- | --- | --- | --- | --- | --- |
|  |  | **Hazard ratio**  **(95% CI)** | **p-value** | **Hazard ratio**  **(95% CI)** | **p-value** |
| Type of Surgery  Pancreatoduodenectomy  Distal pancreatectomy  Total pancreatectomy | 220 (69.8)  52 (16.5)  43 (13.7) | 1 (ref)  1.306 (0.913-1.868)  1.277 (0.870-1.874) | -  0.143  0.213 |  |  |
| Vascular resection  No  Yes | 233 (74.0)  82 (26.0) | 1 (ref)  **1.435 (1.067-1.931)** | -  **0.017** |  |  |
| R-status  R0  R1 | 197 (62.5)  118 (37.5) | 1 (ref)  **1.717 (1.309-2.251)** | -  **<0.001** | 1 (ref)  **1.350 (1.001-1.821)** | **-**  **0.049** |
| Lymph-vascular invasion  No  Yes | 100 (31.7)  215 (68.3) | 1 (ref)  **1.566 (1.161-2.113)** | -  **0.003** |  |  |
| Perineural invasion  No  Yes | 69 (21.9)  246 (78.1) | 1 (ref)  **1.707 (1.201-2.426)** | -  **0.003** |  |  |
| Peripancreatic fat invasion  No  Yes | 94 (29.8)  221 (70.2) | 1 (ref)  **1.484 (1.090-2.021)** | -  **0.012** |  |  |
| T- Status  T1  T2  T3  TX | 127 (40.3)  146 (46.3)  18 (5.7)  24 (7.6) | 1 (ref)  **2.211 (1.629-2.999)**  **3.701 (2.134-6.420)**  **1.963 (1.192-3.235)** | -  **<0.001**  **<0.001**  **0.008** | 1 (ref)  **1.550 (1.093-2.197)**  **2.200 (1.202-4.018)**  1.687 (1.003-2.837) | **-**  **0.014**  **0.011**  0.053 |
| N- Status  N0  N1  N2 | 124 (39.4)  111 (35.2)  80 (25.4) | 1 (ref)  1.367 (0.985-1.898)  **3.111 (2.220-4.359)** | -  0.062  **<0.001** | 1 (ref)  1.127 (0.791-1.605)  **2.244 (1.517-3.317)** | -  0.509  **<0.001** |
| Postoperative complications  No  Yes | 135 (42.9)  180 (57.1) | 1 (ref)  1.208 (0.921-1.585) | -  0.173 |  |  |
| Severe postoperative complications (CD ≥ 3)  No  Yes | 263 (83.5)  52 (16.5) | 1 (ref)  1.362 (.962-1.928) | -  0.082 |  |  |
| Adjuvant treatment  No  Yes  Unknown | 96 (30.5)  215 (68.3)  4 (1.3) | 1 (ref)  0.883 (0.659-1.185)  - | -  0.408  - |  |  |

CD, Clavien Dindo

^*^Adjusted for clinical, radiologic and Ca 19.9 values as for in Table 3
